# New Operation for the Extraction of Cataract

**Published:** 1872-09

**Authors:** A. W. Calhoun

**Affiliations:** Vienna, Austria


### FOREIGN CORRESPONDENCE.

### NEW OPERATION FOR EXTRACTION OF CATARACT.

BY A. W. CALHOUN, M.D.

While new and important discoveries are being constantly made in other branches of medicine, the eye—in structure and function, one of the most complicated organs in the whole system—can justly claim, for the correction of its many diseases and anomalies, vaster and more satisfactory improvements, in the last few years, than any other special portion of the body. Much of the best talent in the profession is exclusively engaged in the scientific study and practice of all that pertains to the multitude of affections, both of an operative and other character, and also of the difficult, and at first sight obscure, derangements of accommodation and refraction. Thanks to their zeal, their efforts have not been unavailing. Especially prolific have quite recent years been in bringing before the profession different operations, from different ophthalmologists, for the rapid and safe removal from the eye of a cataractous lens. One of the most recent and prominent amongst these, is that coming from Professor Weber, of Darmstadt, in Germany, and known as "Weber's Method of Extraction."

Every surgeon, who is acquainted with the different operations upon the eye, knows what the "Grâefe's Peripheral Linear Extraction" is; for since its introduction by its celebrated author, more than fifteen years ago, it has almost entirely absorbed all other modes, being applicable to a variety of species of cataract, each of which required a separate and distinct operation previous that proposed by Grâefe. But although of such general adoption from its introduction to the present moment, and of such favorable results in the large per centage of recoveries, still even this favorite operation has a few defects—or, rather, is accompanied by a few unpleasant consequences—the most important of which is the tendency to a loss of vitreous humor during the removal of the lens. To remedy these difficulties, and to retain all the good of Gràefe's extraction, Weber proposed, in 1867, the plan now bearing his name, and which is at this time attracting much attention, and being put upon trial in many of the German schools, and, up to the present, with brilliant success.

The knife suited to this operation, and used by Weber himself, is spear-shaped, and bent at an obtuse angle at the junction of the blade and handle—in short, resembling the ordinary curved iridectomy knife, with the exception that it is broader at the base, and possibly longer from base to point. No other particular instrument is required for the performance of this operation, which is made as follows:

After separating the lids, either by means of an eye-speculum, or through the fingers of an assistant or otherwise, the operator fixes the ball with one hand by grasping, with suitable fixation forceps, a fold of the conjunctiva below the cornea, thus enabling him to draw the eye downward, and enters the knife held in the other hand from above into the anterior chamber through the "limbus," the conjunction of the schlera and cornea. Raising the hand, and thereby the handle of the instrument, so as to throw the point forward, and prevent its entanglement in the iris, it is gently advanced forward till it reaches and almost touches the lower edge of the chamber, when the blade is carefully withdrawn, the wound being now of sufficient breadth to admit of the easy passage of the lens. The iridectomy and opening of the capsule are next made in the usual manner, as in the linear extraction; and the fixation being now resumed from below, the dislocation of the upper edge of the lens forward, and finally into the anterior chamber through the opening in the iris, is induced by slight pressure from above and near the anterior portion of the schlera, by some suitable instrument, and like pressure from below by means of the fixation forceps; then, by assisting its exit by lightly drawing the instrument over the cornea from below toward the wound, the lens escapes readily out of the bulbus. This pressure must of course be made with great care—the object being simply to tilt the upper edge of the lens into the anterior chamber, and then, by the pressure from below, to help it through the external wound.

This cut through the external coat of the eye constitutes the important difference between this and Gräefe's operation. Every portion of the wound falls in the corneo-schlerotic junction; and after the removal of the lens, the opening closes with exactness in its entire extent, and no uneasiness need be felt as to the gaping of the cut surfaces. The greatest advantage it possesses over Gräefe's is the almost absolute exemption from loss of vitreous humor. As before remarked, this dangerous consequence is one of the few disadvantages connected with the above operation, and is very nearly completely avoided by Weber's, for out of nearly one hundred cases in the wards of Prof. Arlt, vitreous humor has been lost in only one, and then but to a slight extent. It is true that a small loss of this humor can often be surprisingly well-borne by many eyes; yet the dangers are too well known, and the sad results are of too frequent occurrence, to permit of the least underrating, or to call for any further mention here. Although the ultimate success of Gräefe's operation has everywhere been flattering in the highest degree, and far exceeding all other previous methods, still very often, in the unsuccessful cases, can the failure be attributable to this greater or less escape of vitreous humor. The shape and position of the wound, etc., made as proposed by Weber, avoids this with tolerable certainty.

Another advantage is the ease with which the operation is made, no more difficulty being attached to its various steps than to an ordinary iridectomy; for not only the cut and the other points necessary to be carried out are very similar, but also the instruments are very nearly the same as those used in iridectomy. The maneuvre for the removal of the lens itself is the same in both operations.

Still another recommendation, and not unfrequently a consideration of the greatest importance, is the shortness of time required, in favorably progressing cases, between the operation and the dismissal from treatment. On the second day is the patient allowed to sit up in his bed, and on the third, to occupy his chair; and in a few instances, they have been sent to their homes after the expiration of eight days, the case having been brought to a good end. The length of time required for treatment in bed after the Grâefe operation is one of its great disadvantages; for mostly, under the most favorable progress, it is not considered safe to allow the patient to leave his bed under five or six days; and the remaining items of the after-treatment demand a proportionately long time, even after he is permitted the limits of his room.

Where no untoward event interferes, the wound heals rapidly and kindly, often leaving no cicatrix whatever, or any trace of the cut—the limbus at this point appearing as free and normal as in any other portion of its untouched circumference. After the linear extraction, as well as after others, very frequently, a small cyst forms upon the cicatrix, which, before becoming perfectly hard and resisting, gives way under the pressure from the fluids or humors of the eye, and to greater or less extent is formed a small, often almost transparent bladder, situated immediately on or over the wound. So far as observed here, this cyst has appeared comparatively seldom after any of the operations performed in accordance with Weber's plan. So far as vision is concerned, such a new formation is of no consequence; but the patient is somewhat troubled by its presence, the upper lid in its movements rubbing to and fro over it; and also is it a conspicuous and not very beautiful point, which is not only seen when the eye is cast in certain directions, but can frequently be detected by the slight projection of that portion of the lid with which it comes in contact.

There is one hindrance to the complete success of the operation, viz: that considerable portions or remnants of the lens often remain behind after the extraction; and these remnants are not unfrequently so tenacious in their attachments as to resist all the usual or reasonable means practiced for their dislodgment and removal from the anterior or posterior chambers. Fortunately, however, these pieces of lens amount to nothing more than that they for a time prevent the pupil from being as free as it should be after a cataract operation. They are absorbed in a short while, creating no trouble whatever by their existence in either of the chambers, and disturb the vision but little, as they occupy only a small part of the pupil, the other portion being clear for the entrance of rays of light.

In almost all cataract operations by extraction, with the external wound in whatever part of the cornea, the healing takes place in such a manner as to a greater or less degree disturb the exact and natural course of the cornea, which curvature is absolutely essential to the perfect or emmetropic vision of the individual. Good seeing does not, of course, depend alone upon this natural condition of the cornea, but also upon the normal state of the other refracting media of the eye, which still remain after the removal of the lens; but with the cornea deranged in any of its curves, and to any extent, that anomaly of refraction known as astigmatism is easily produced, and which, when "regular," mostly allows correction by means of suitable glasses at the same time we supply the defect of the missing lens; but when "irregular," and of a high degree, is but seldom entirely relieved by the use of any glass.

This difficulty, also, is less frequently met with after Weber's extraction, where the wound is not made through the cornea itself, properly speaking, but in the very conspicuous circle or limbus formed by the junction of the cornea with the schlera. It must be mentioned, however, that astigmatism resulting from cataract operation is, as a rule, so slight, that no correction by means of cylindrical glasses is necessary; yet that it does take place, and sometimes to an injurious extent, can be proven to any one who will trouble himself to test the point upon different cases.

The operation as now performed by Prof. Arlt differs slightly from that originally proposed by Prof. Weber, but the same idea is kept in view throughout. Instead of making the wound in the lower part of the limbus or circle formed by the junction of schlera and cornea, and generally without iridectomy (Weber's method), Arlt operates in the upper portion, and always with iridectomy, from the fact that the upper cut is attended with better consequences, and probably more easily made; and combines the iridectomy to ward off subsequent iritis, which occurred in his first few cases in which no iridectomy was made, by the lens pressing too much upon, and thus affording too great a source of irritation to the iris in its passage through the pupil into the anterior chamber, previous to its exit from the eye.

As yet, strange to say, no statistics of the number of operations, or of the per cent. of recoveries, have been published, although it has been extensively practiced in many of the German hospitals for the past six or eight months more especially, and the only data we have are the verbal reports from the other schools, and the private statistics kept of the cases operated upon by Prof. Arlt.

Since 1st April of this year (1872), between ninety and one hundred cases have been operated upon by Weber's method in the eye wards under the charge of Prof. Arlt, and with only a loss of six per cent.; and one or two of the cases classed amongst the lost may possibly be partially restored by some additional operation, since the good termination of the first was prevented by the occurrence of irido-cyclitis, which, in addition to its other damaging effects, also occludes the pupil; and where no serious damage has been done in the interior of the bulbus, a new iridectomy may perhaps be of some service in giving at least a small amount of vision to the patients.

At present, so far as can be ascertained from different sources, the results of Weber's operation equal those of Grâefe's, and even possess some advantages over it; but the latter is not soon to be superseded, since it is adapted to such a variety of cataracts, and with equally good results in each. Yet if the general statistics, which must sooner or later be published, give the same proportionate success as has been communicated by individual operators, the operation is destined to stand side by side with the linear extraction, and become one of the standard methods for the relief of cataract.

